# On frailty and accelerated aging during SARS-Cov-2: senescence

**DOI:** 10.1007/s40520-023-02364-4

**Published:** 2023-03-20

**Authors:** Ursula Müller-Werdan, M. Cristina Polidori, Andreas Simm

**Affiliations:** 1 Department of Geriatrics and Medical Gerontology, Charité – Universitätsmedizin Berlin and EGZB, Berlin, Germany; 2 Department II of Internal Medicine and Center for Molecular Medicine Cologne, Ageing Clinical Research, University of Cologne, Faculty of Medicine and University Hospital Cologne, Cologne, Germany; 3 CECAD, University of Cologne, Faculty of Medicine and University Hospital Cologne, Cologne, Germany; 4 Clinic for Cardiac Surgery, University Hospital Halle (Saale), Martin-Luther-University Halle-Wittenberg, Halle (Saale), Germany

**Keywords:** Aging, Biological Age, COVID-19, Coronavirus, Frailty, Inflammaging, Senescence

## Abstract

The COVID-19 pandemic is a burden for the worldwide healthcare systems. Whereas a clear age-dependent mortality can be observed, especially multimorbid and frail persons are at an increased risk. As bio-functional rather than calendrical age is in the meanwhile known to play a crucial role for COVID-19-related outcomes, aging-associated risk factors, overall prognosis and physiological age-related changes should be systematically considered for clinical decision-making. In this overview, we focus on cellular senescence as a major factor of biological aging, associated with organ dysfunction and increased inflammation (inflammaging).

## Introduction

After more than 3 years of severe acute respiratory syndrome-corona virus 2 (SARS-Cov-2) pandemic, over 753 million confirmed cases and 6.8 million deaths have been reported globally (WHO Coronavirus Situation Report, February 2023). In the meanwhile, it is well established that deaths from Coronavirus Disease 2019 (COVID-19) occur predominantly among seniors and that COVID-19 appears to be particularly dangerous for older men, essentially sparing other vulnerable age groups usually affected by similar viruses. Although the occurrence of new variants and the vaccine have substantially influenced the course of the pandemic, age is the ultimate risk factor for mortality in COVID-19, with rates significantly and progressively enhanced at the age 80 + and 70 + . In the past recent months, however, evidence has accumulated that *frailty*—intended as surrogate marker of *biological age*—not per se *age*—intended as *chronological age*—is the ultimate risk factor for COVID-19 mortality, and we have proposed that the main reason for high SARS-CoV-2 lethality in advanced age is the impact of COVID-19 on overlying dimensions of the organism [1]. Indeed, although we currently lack a gold standard as a metric for the measurement of biological age [2], several most important biological mechanisms of aging have been identified as *hallmarks* or *pillars* of aging [3].

Evidence strongly suggests that biological aging—like frailty—is multifactorial and is grounded not only on several diverse interactions among the hallmarks of aging, but is multidimensional and influenced by functional and psychosocial factors beyond organ illness [4, 5]. Aging is per definition an inter-individually and intra-individually heterogeneous process, and age-related changes including those of organs do not occur at the same rate and to the same extent in all older persons [6]. Accordingly, there are many cases of recovery after COVID-19 severe respiratory distress in the multimorbid oldest old, including cases of centenarians who survived the Spanish flu and the Second World War [7]. Like being 80 years old or older cannot be the unequivocal basis for the definition of being a geriatric patient, this chronological age threshold should not be used for triaging, stratification and, in the case of COVID, for denying resource allocation in the name of “clinical reasonableness” or “soft utilitarian” approach [1]. Many active 80-plus persons worldwide have a remaining, often disability-free, life expectancy of more than 9 years (https://www.ssa.gov/oact/STATS/table4c6.html).

In addition to the knowledge that COVID-19 lethality is highest in frail persons beyond chronological age and comorbidity, there is increasing evidence that COVID-19 accelerates the pace of intrinsic aging and therefore biological age and frailty [8]. In this overview, we summarize evidence showing the predominant mechanisms of this effect.

## On biological hallmarks and phenotypes of aging and COVID-19

**Fig. 1:**
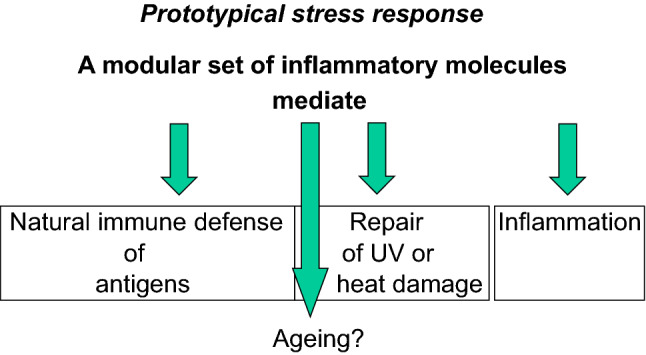
Prototypical stress response is a modular reaction to both antigens and chemical or physical stimuli challenging integrity of the organism, with an up-regulation of evolutionarily conserved mediators of natural immunity. Thus, there is an equivalence of antigen and other stimuli in evoking this pattern of mediators, which may also account for inflammaging

The traditional medical paradigm based on the biological model of disease assumes that illnesses have typically one cause and one pathophysiological mechanism upon which their diagnosis and therapy fund significance and success. With the increasing complexity of clinical pictures and disease course due to multimorbidity and geriatric syndromes, however, the traditional paradigm is strongly challenged. This challenge has emerged powerfully during COVID-19, which involves cellular, tissue, organ-systemic and functional factor—from host receptors of SARS-CoV-2 in the cell to the radiologic and histopathologic alterations observed across COVID-19 disease severity stages—involved in all well-established pathways of the physiological aging process. These include senescence-related receptors CD26 and angiotensin-converting enzyme 2 as well as inflammation-related mechanisms, immunological alterations, hypoxia-related redox signaling changes, and endothelial dysfunction [For review, see 1]. Some main examples include age-related changes such as inflammaging and immunosenescence which appear to pave the way to SARS-CoV-2 pathogenicity mechanisms—the “cytokine storm”, the senescent endothelium which predisposes to the vascular manifestations of COVID-19, and the senile lung which impacts on COVID-19 diagnosis [1]. It seems clear that an infection with SARS-Cov-2 can induce in cells a prototypical stress response (Fig. 1).

As SARS-CoV-2 is detected in pneumocytes, less in macrophages and bronchiolar epithelial cells, and is associated with hemorrhagic necrosis and lymphocyte depletion in lymph nodes and spleen, lymphocytopenia [9] is displayed. The latter manifests as both less production of new naive lymphocytes and loss of functional competence of memory cell populations, which are typical age-related changes [6]. Correlated to these important phenotypes, diversity and complex regulatory paths of the immune network are progressively lost with advancing age, loss which represents an established factor associated with increasing frequency and severity of breakthrough infections in older persons [10, 11]. As a consequence of the complexity and multifactoriality of aging, many of the age-related changes at the biological level synergistically contribute to higher SARS-Cov-2 pathogenicity and susceptibility of the host to this pathogen. A typical example is that the detrimental effects of the COVID-19- related overproduction of early response pro-inflammatory cytokines resulting in the *cytokine storm* can be sharply enhanced by the vascular hyperpermeability characteristic of the senescent endothelium. In turn, the cytokine storm can cause per se vascular hyperpermeability, which reveals particularly dangerous in the aged, already dysfunctional endothelium [1].

Age-related endothelial dysfunction with tendency to vessel rupture and multiorgan bleeding on one hand and thrombosis commonly observed in small vessels of severely ill COVID-19 patients in extrapulmonary organs free from coronavirus suggest alternative mechanisms beyond viral infection, such as hypoxemia and ischemia. These are also well-established age-related changes and hypoxemia-related cascades are associated with oxygen deficiency and increased generation of reactive oxygen species (ROS) occurring with increasing age [12, 13]. As another crucial example of multifactoriality and complexity of aging, metabolites generated by redox reactions have the capacity to modify macromolecules over time, and in turn this cumulative macromolecular damage contributes to many mechanisms of aging, including sustained ROS-induced endothelial dysfunction and microthrombotic phenomena [6]. Accordingly, a spectrum of substances with diverse mechanisms of action like natural products, probiotics, and nutrients with antimicrobial, antiviral, analgesic, anti-inflammatory, and antiproliferative activities have shown some efficacy in improving immunity against viral infections through inhibiting viral cell entry, replication, and modulation, and therefore in counteracting COVID-19-related symptoms [1, 14].

## Focus on senescence

As mentioned above, among the *hallmarks of aging*, one of the main drivers of COVID-19 lethality is senescence (for the purpose of this contribution, “cellular senescence” is considered pleonastic). Immunosenescence with low self-renewal capacity of specialized immune cells like lymphocytes and chronic low-grade inflammation might be a facilitating factor of SARS-CoV-2-induced pathogenicity [11]. Senescence is a complex stress response that causes an essentially irreversible arrest of cell proliferation, resistance against apoptosis, and the development of a multicomponent senescence-associated secretory phenotype (SASP) [15].

Research in animal models and some initial data in humans suggest the hypothesis that the typical pro-inflammatory state of aging is in large part sustained by the accumulation of senescent cells and the spilling in the blood of SASP including cytokines and chemokines. Systemic, sustained low-grade chronic inflammation due to persistent tissue damage, environmental stressors, unhealthy lifestyle, and social and psychological stress represents a modular response of natural immunity and, indeed, is associated with the risk of developing many chronic diseases—insulin resistance, CVD, osteoarthritis, chronic obstructive pulmonary disease (COPD) and neurodegenerative processes—all characterized by the accumulation of senescent cells [16]. Dysfunction in many of the hallmarks of aging often converge into a pro-inflammatory response and exogenous compounds such as bacteria or viral fragments or endogenous chemicals have been shown to interact with pattern recognition receptors (PPRs) expressed on the cell surface and in the cytoplasm. PPRs like Toll-like receptors, NOD-like receptors and aryl hydrocarbon receptors trigger inflammatory responses, thereby inducing inflammaging [11]. In addition, patients with different age-related chronic conditions do display high circulating levels of several inflammatory biomarkers, including CRP, IL-6, IL-18, and TNF, while IL-6 serum levels have been also shown to predict disability and frailty [reviewed in 17]. Furthermore, high blood IL-6 values have been found to be associated to lower walking speed and the risk of developing disability overtime was observed to increase linearly for IL-6 levels higher than 2.5 pg/ml [17].

**Fig. 2:**
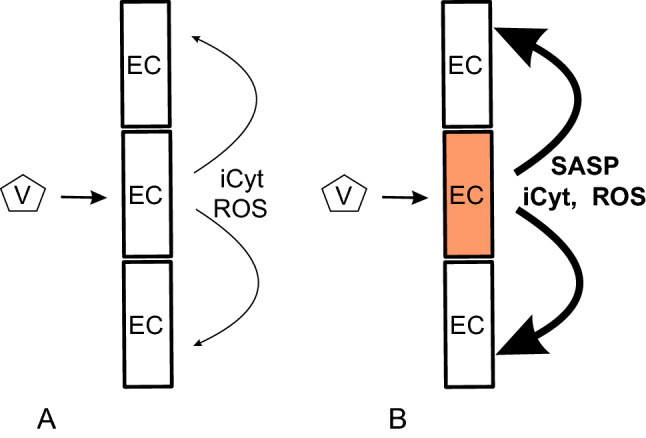
Vessel dysfunction may depend on the accumulation of senescent endothelial cells: **A** Virus infects cells which induce inflammatory cytokines (iCyt) and ROS; **B** Senescent cells (red) enhance the tissue response and increase inflammation

In addition to inflammaging, garbaging, senescence and SASP, the individual’s history of exposure to certain microorganisms (eg. HIV and CMV) or antigens—immunobiography—may influence the degree and characteristic of the inflammatory response to various stimuli, SARS-Cov-2 lethality, inflammging itself and frailty. For instance, immunobiography may mediate the chronic inflammation and abnormal production of cytokines (TNF, IL-1, and IL-6) as well as altered immune T-cell response characterizing metabolic syndrome and adiposity, in turn risk factors for poor outcomes during COVID-19 [1]. Nguyen et al. found that the senescence phenotype is robustly upregulated in most SARS-CoV-2-infected patients [18]. Schmidt et al. proposed that SASP-driven spreading of cellular senescence can further amplify the SASP by increasing the burden of senescent cells [19]. For this reason, the observation that prognostic factors for mortality from COVID-19 are similar to those that have been found associated with a high risk of chronic inflammation—like older age, male sex, obesity, smoking, cardiovascular diseases—has prompted the suggestion that COVID-19 might induce accelerated aging and frailty across a wide range of severity [1, 8, 10, 16]. Such a prototypical inflammatory stress response characterizes individuals of old age with a, however, wide variation with the general population [20–22]. The progressive chronic basal systemic inflammation does not necessarily go along with clinical signs of illness, but may compromise defense against microbes or noxious agents by desensitizing natural immune response. These alterations forming part of the physiological aging process of the immune system as immunosenescence are seen in both vertebrates and non-vertebrates [23]. Claudio Franceschi [24] pointed out that lifelong antigen stress including encounters with severe pathogens (“inflammatory/ pathogen burden”) may account for interindividual diversity in aging, which is referred to as “garbaging” in view of a decline in autophagy of accumulating metabolic waste products [11].Judith Campisi and her group found that these senescent cells can go into permanent cell cycle arrest: thereby, on the one hand, the proliferation of damaged cells at risk of neoplastic transformation is stopped, however, on the other hand, these cells can accumulate in the tissues, get dysfunctional and evoke a SASP further promoting inflammation [25]. Therefore, preexisting senescent endothelial cells may accelerate the vascular inflammation and dysfunction (Fig. 2). Drugs eliminating these senescent cells may slow aging and are currently being tested in humans [26]. SASP has been characterized largely as a myriad of cytokines, chemokines (CXCLs), growth factors, miRNAs and proteases that participate in embryogenesis, wound healing, inflammation, and many age-related pathologies. Recently, several candidate biomarkers of senescence were identified that overlap with aging markers in human plasma, including Growth/differentiation factor 15 (GDF15), stanniocalcin 1 (STC1), and serine protease inhibitors (SERPINs) [27]. Moreover, SASP proteins were found to be positively associated with age, frailty, and adverse postsurgery outcomes [28]. In this study, a panel of 7 SASP factors composed of growth differentiation factor 15 (GDF15), TNF receptor superfamily member 6 (FAS), osteopontin (OPN), TNF receptor 1 (TNFR1), ACTIVIN A, chemokine (C–C motif) ligand 3 (CCL3), and IL-15 predicted adverse events markedly better than a single SASP protein or age [28]. While lipid components of the SASP are understudied, senescent cells were recently shown to activate the biosynthesis of several oxylipins that promote segments of the SASP and reinforce the proliferative arrest. Notably, senescent cells appear to synthesize and accumulate an unstudied intracellular prostaglandin, 1a,1b-dihomo-15-deoxy-delta-12,14-prostaglandin J2. Released 15-deoxy-delta-12,14-prostaglandin J2 is suggested as a biomarker of senolysis in culture and in vivo. This and other prostaglandin D2-related lipids were observed to promote the senescence arrest and SASP by activating RAS signaling [29], suggesting a new method to detect senolysis.

These findings, facilitating the identification of proteins characteristic of senescence-associated phenotypes, may help the cataloging of potential senescence biomarkers to assess the burden, originating stimulus, and tissue of origin of senescent cells in vivo in COVID-19.

## Future perspectives and conclusions

In Europe, the number of older adults aged 60 years and over is set to double between 2002 and 2025, to reach a population of 1.2 billion in 2025, with fastest growth expected among those aged ≥ 85 years. Population projections suggest that the EU-27 old-age dependency ratio will continue to climb and will peak at 56.7% by 2050, when there will be fewer than two persons of working age for each older person. Accordingly, there will be half a million centenarians in the EU-27 by 2050 (http://ec.europa.eu). A key public health challenge is to ensure citizens remain healthy and disability-free for as long as possible, but the increase in life expectancy and global population aging in the past 50 years have resulted in substantial challenges to societies and health care systems. These have been maximized by COVID-19. Disability outweighs multimorbidity in regard to associations with adverse health outcomes in older adults, as its development is a multifactorial process that involves biopsychosocial factors. Frailty, which precedes disability and represents the transition between healthy and unhealthy states, has been well recognized as an age-related vulnerability resulting from impaired homeostasis, reduced physiological reserve and various stressors also related to biopsychosocial domains [30]. Independent of definitions, frailty increases the risks for falls, fractures, unplanned hospitalizations, physical dependence, nursing home placement or mortality of older adults. In most literature, frailty refers to physical frailty because of its direct relationship with physical disability. In this sense, the physical phenotype of frailty can be defined as a geriatric syndrome. However, the multidimensional construct of frailty may be overlooked if only the physical domain is addressed. Similarly to the huge limitation delivered to clinical care if frailty is only considered of biological nature [31], psychosocial frailty conceptualized to address the state of insufficient participation in social networks and the perception of a lack of contacts and cognitive ability maybe also limited in overview [32–35]. Frailty, as the extreme end of accelerated aging immediately preceding disability, is much more the very core of Ageing Medicine and Geriatrics. The mission of the latter is, therefore, the identification of measures of accelerated aging, which is substantially more difficult in the pre-clinical phase, when older persons are still cognitively and functionally intact and are not affected by overt multimorbidity. Within the attempts to measure biological aging for a clinical context [30, 36], the accumulation of cellular senescent cells and the contribution of these cells by their associated SASP to an increased low-grade systemic inflammation seems to be a reasonable target [37]. While COVID-19 unmasked possible critical mechanisms of senescence induction and acceleration during aging, focused search on biology underneath accelerated aging, on inflammaging for the development of chronic diseases and in particular on entangled mechanisms of biopsychosocial resilience are urgently needed.
